# A novel pathophysiologic link between upper and lower airways in patients with chronic rhinosinusitis: Association of sputum periostin levels with upper airway inflammation and olfactory function

**DOI:** 10.1016/j.waojou.2019.100094

**Published:** 2020-01-06

**Authors:** Yoshihiro Kanemitsu, Motohiko Suzuki, Kensuke Fukumitsu, Takamitsu Asano, Norihisa Takeda, Yoshihisa Nakamura, Yoshiyuki Ozawa, Ayako Masaki, Junya Ono, Ryota Kurokawa, Jennifer Yap, Hirono Nishiyama, Satoshi Fukuda, Hirotsugu Ohkubo, Ken Maeno, Yutaka Ito, Tetsuya Oguri, Kenji Izuhara, Masaya Takemura, Akio Niimi

**Affiliations:** a Department of Respiratory Medicine, Allergy and Clinical Immunology, Nagoya City University Graduate School of Medical Sciences, Nagoya, Japan; b Department of Otorhinolaryngology, Nagoya City University Graduate School of Medical Sciences, Nagoya, Japan; c Department of Radiology, Nagoya City University Graduate School of Medical Sciences, Nagoya, Japan; d Department of Pathology, Nagoya City University Graduate School of Medical Sciences, Nagoya, Japan; e Shino-Test Corporation, Sagamihara, Japan; f The Division of Medical Biochemistry, Department of Biomolecular Sciences, Saga Medical School, Saga, Japan

**Keywords:** Asthma, Chronic rhinosinusitis, Olfactory dysfunction, Eosinophils, Periostin, Fractional exhaled nitric oxides, AHR, airway hyperresponsiveness, ATS, American Thoracic Society, COPD, chronic obstructive pulmonary disease, CRS, chronic rhinosinusitis, CRSsNP, CRS without nasal polyps, CRSwNP, CRS with nasal polyps, CT, computed tomography, ESS, endoscopic sinus surgery, ERS, European Respiratory Society, FeNO, fractional nitric oxides, GINA, Global Initiative for Asthma, HPF, high-power field, IL, interleukin, LMS, Lund-Mackay score, NPs, nasal polyps, SNOT-22, Sinonasal Outcome Test-22, Tukey Kramer HSD, Tukey Kramer honestly significant difference

## Abstract

**Background:**

Chronic rhinosinusitis (CRS) and asthma are collectively called unified airway diseases. Periostin has been implicated in the pathophysiologic link of these conditions but only by serum measurements. We sought to investigate sputum levels of periostin and their association with upper airway inflammation and olfactory function in CRS patients.

**Methods:**

We prospectively recruited 56 CRS patients who underwent endoscopic sinus surgery (20 with and 36 without comorbid asthma), and 28 healthy controls between October 2015 and December 2017. Lower and upper airway indices such as sputum periostin levels and eosinophil and neutrophil counts, exhaled fractional nitric oxide (FeNO) levels, and olfactory function were evaluated in the three groups. Radiological severity of CT images and tissue eosinophilia of surgical specimens were also assessed in the CRS patients.

**Results:**

Sputum periostin levels were highest, and olfactory function was most impaired, in the CRS patients with comorbid asthma, followed by those without asthma and controls in this order. CRS with asthma group showed higher sputum eosinophils and FeNO levels than the other two groups, while CRS patients without asthma showed significantly higher neutrophils in sputum than the other two groups. When confined to CRS patients, olfactory dysfunction was correlated with sputum eosinophil counts. Eosinophil counts of nasal polyps showed a significant positive correlation with sputum periostin and FeNO levels. Radiological severity of CRS was correlated with sputum eosinophil counts and FeNO levels.

**Conclusions:**

Periostin levels and inflammatory cells such as eosinophils and neutrophils in the lower airways are increased in patients with CRS, suggesting the presence of mutual interactions between upper and lower airways even if asthma does not coexist. Olfactory dysfunction and eosinophilic nasal polyps may be potential indicators of Th2-driven inflammation in the lower airways.

**Trial registration:**

This study was registered on the UMIN Clinical Trials Registry (Registry ID UMIN000018672).

## Introduction

There are close interactions between chronic rhinosinusitis (CRS) and asthma because upper and lower airways are continuous from nasal cavity to the lung parenchyma and functionally linked; they are often collectively called “unified airway diseases”. Indeed, it has been reported that 41–51% of patients with asthma have comorbid CRS,^1^ and asthma coexists in 20–31.9% of patients with CRS according to epidemiologic surveys.^2^ Furthermore, CRS is associated with the development of late-onset asthma^3^ and the exacerbations and poor control of asthma.^1^^,^^4^ Thus, CRS is linked to the pathophysiology of asthma.

Previous studies have demonstrated the interaction between upper and lower airways inflammation. Acute allergen exposure conducted out of hay fever seasons into nasal cavity could facilitate the infiltration of eosinophils in nasal and bronchial mucosa in patients with seasonal allergic rhinitis but without asthma.^5^ Rhinitis patients, irrespective of the presence or absence of asthma, also showed marked infiltration of eosinophils in nasal and bronchial tissues when compared to subjects without rhinitis.^6^ Furthermore, marked eosinophilia in nasal mucosa was also present in patients with asthma even if they did not have concomitant rhinitis.^7^ In patients with severe asthma, Th2-driven biomarkers such as blood and sputum eosinophils and fractional exhaled nitric oxide (FeNO) were associated with radiological severity of concomitant CRS. More severe CRS was also associated with higher functional residual capacity and lower carbon monoxide diffusing capacity.^8^ These findings indicate the existence of naso/sino-bronchial crosstalk between upper and lower airways.

Periostin, a 90-kD extracellular protein which is mainly produced by fibroblasts in response to interleukin (IL)-4 and IL-13, is a notable biomarker which reflects the pathophysiology of both asthma and CRS. It acts as a matricellular protein, and it is involved in the chronicity of allergic inflammation. In patients with asthma, serum periostin levels were higher when patients suffered from comorbid CRS.^9^ The increase of periostin was prominent when patients had nasal polyps (NPs) with olfactory dysfunction.^10^ We have also demonstrated that serum periostin is a sensitive biomarker to detect comorbid CRS with NPs (CRSwNP) in patients with asthma.^11^ Therefore, periostin would be involved in the pathophysiology of unified airway diseases. Also, sputum periostin levels reflect fixed airflow limitation^12^ and sputum eosinophilia^13^^,^^14^ of severe asthmatics with Th2-high phenotypes^12^^,^^13^ and poorly controlled asthma.^14^ However, the relevance of sputum periostin levels to the pathophysiology of unified airway diseases remains unknown.

In this study, we sought to examine periostin levels in the lower airways with induced sputum samples in CRS patients as compared to healthy subjects. CRS patients were subdivided into those with or without comorbid asthma. Other biomarkers of lower airways (sputum eosinophils and neutrophils, and FeNO) were also compared among the three groups. Association of sputum periostin levels with upper airway indices (olfactory function, CRS symptoms, resected sinus and NP tissue eosinophilia, and radiological CRS severity) were also analysed, along with sputum cells and FeNO levels.

## Methods

### Patients and diagnosis

We prospectively recruited CRS patients and those with comorbid asthma who visited our hospital to undergo endoscopic sinus surgery (ESS) between October 2015 and December 2017. Patients were diagnosed as having asthma when they complained of asthma-related symptoms such as cough, dyspnoea, chest tightness and/or wheezing with the presence of clinical reversible airway obstruction or airway hyperresponsiveness (AHR) to inhaled methacholine.^15^ AHR was measured by continuous inhalation of 10 doubling concentrations of methacholine (49–25,000 μg/mL) (Astograph®; Chest, Tokyo, Japan).^16^^,^^17^ The total cumulative dose of methacholine at the end of inhaling the highest concentration was 50 units. Subjects inhaled methacholine until maximum methacholine concentrations or 2 folds of baseline respiratory resistance (Rrs) values determined by 1 minute inhalation of saline before the initiation of inhaled methacholine, followed by salbutamol inhalation for 2 minutes. Dmin, the cumulative dose of inhaled methacholine at the inflection point at which Rrs began to increase, represents airway sensitivity. AHR was considered positive if Dmin was ≤12.5 units.^17^ All patients with asthma were confirmed the presence of AHR to inhaled methacholine in our hospital (n = 18) and/or clinical airway reversibility with the response to β_2_ agonists for their symptoms previously. The diagnosis of CRS was made according to radiological and endoscopic findings with physical symptoms lasting for 12 weeks or longer.^18^ Endoscopic and radiological examinations show NPs, purulent mucus and submucosal edema in the middle meatus or anterior ethmoid cavity. When these endoscopic and/or radiological features were applicable in combination with two or more physical symptoms (mucopurulent drainage, nasal obstruction, facial pain and/or impairment of smelling), patients were diagnosed as having CRS.^18^ CRS were stratified according to the presence or absence of NPs (CRSwNP and CRSsNP, respectively).^18^ All patients underwent medical check by both asthma and otorhinolaryngology specialists to evaluate the existence or condition of asthma and CRS, and the indication of ESS. Healthy volunteers who had no history of either upper or lower airway diseases were also enrolled as controls between October 2015 and December 2017. Participants were excluded if they smoked currently or quit smoking within the past six months, had other pulmonary diseases including chronic obstructive pulmonary disease (COPD), took oral corticosteroids, or experienced acute respiratory infection within 4 weeks prior to enrollment. This study was approved by the Ethics Committee of our hospital (1165) and was registered on the UMIN Clinical Trials Registry (Registry ID UMIN000018672). Written informed consent was obtained from all participants.

### Measurements

All participants underwent spirometry, FeNO measurement, olfactory function test and sputum induction, and answered the Sinonasal Outcome Test-22 (SNOT-22). Only one CRS patient with comorbid asthma failed to conduct the NO measurement because of apparatus failure. Furthermore, all CRS patients underwent blood analyses [eosinophils, total IgE, and specific IgE antibody against 9 aeroallergens; house dust mite, cat, dog dander, Japanese cedar pollen, mixed Gramineae pollens (orchard grass, sweet vernal grass, Bermuda grass, Timothy grass and reeds), mixed weed pollen (ragweed, mugwort, goldenrod, dandelion and oxeye daisy), *Alternaria alternata*, and *Staphylococcus aureus* enterotoxin A and B], sinus computed tomography (CT) scan, and ESS. Patients were considered to have atopic predisposition if one or more specific IgE antibody titer showed ≥0.35 UA/ml. All measurements except for tissue sample collections were conducted before ESS.

### Evaluation of upper airways

Olfactory function was evaluated using the Open Essence method.^19^^,^^20^ Briefly, Open Essence consists of 12 different smelling cards (i.e. condensed milk, cooking gas, curry, cypress wood, India ink, Japanese orange, menthol, wood, rose, sweaty-smelling clothes, perfume, and roasted garlic). Each card was sniffed, and then the odorant was chosen among 4 alternatives in order. The number of correct scores (Open Essence scores ranging from 0 to 12) was calculated. Higher scores represent better sense of smell (i.e. 0 = anosmia, 12 = good sense of smell).

SNOT-22 is a validated questionnaire which is composed of the most 22 CRS-related symptoms regarding quality of life (QoL).^21^ All symptoms range from 0 to 5 (0 = “No Problem’’, 1 = ‘‘Very mild problem’’, 2 = “Mild or slight problem’’, 3 = ‘‘Moderate’’, 4 = ‘‘Severe Problem’’, and 5 = ‘‘Problem as bad as it can be’’). Higher scores represent worse CRS-related QoL and predict good response to ESS.^21^ The reliability and validity of the Japanese version of the original questionnaire, SNOT-20, have already been validated.^22^ We obtained the permission to use it for this study from Professor Jay Piccirillo, Washington University, USA.

The Lund–Mackay score (LMS) was adopted to evaluate radiological severity of CRS. Briefly, scores ranging from 0 to 2 assigned into each of unilateral sides of the maxillary, anterior ethmoid, posterior ethmoid, sphenoid and frontal sinuses (0: no abnormality, 1: partial opacification, or 2: total opacification), and the ostiomeatal complex (0: not obstructed, or 2 obstructed). The total scores range from 0 to 24. Higher scores indicate more severe CRS (i.e. 0 = complete lucency, 24 = complete opacity of all the sinuses).^23^ The LMS was determined by one radiologist who is a specialist of the head and neck regions.

All of ESS were performed, and inflamed sinus tissue samples were taken from 54 patients under general anesthesia by otorhinolaryngology specialists. The remaining 2 patients, all without asthma, underwent resection of NPs only because there was no mucopurulent discharge in sinuses when ESS was performed. NPs were resected from 38 patients. After fixing in formalin and embedding in paraffin, 4-μm paraffin sections were stained with Hematoxylin-Eosin. The number of eosinophils in NPs and sinus tissues per high-power field (HPF, 400 × ) was counted by one pathologist. Radiological and histological evaluations were independently performed under a blinded manner, respectively.

### Evaluation of lower airways

Spirometry was performed to assess prebronchodilator FEV_1_ according to the ATS/ERS recommendations using Chestac-8900 (Chest Corp, Tokyo, Japan).^24^

FeNO levels were measured with an oral expiratory flow rate of 50 ml/s using a Sievers NOA 280i chemiluminescence analyzer (GE Analytical Instruments, Boulder, USA).^19^^,^^25^ In patients with asthma, they withheld taking anti-asthma drugs including ICS 24 hours before the measurement of spirometry and FeNO.

The sputum samples were obtained from 65 participants (16 with and 29 without asthma, and 20 controls) with 15 minute inhalation of 3% saline after 400 μg of inhaled salbutamol.^26^ After counting 400 differential cells, the proportion of neutrophils and eosinophils was calculated. Periostin levels of sputum supernatant were measured with the use of an enzyme-linked immunosorbent assay developed by Shino-Test (Kanagawa, Japan).^9^^,^^11^ SS18A and SS19C, both of which are antibodies against FAS I domains, were used to determine sputum periostin levels.

### Statistics

Data were analyzed using JMP 11.2.0 Start Statics (SAS Institute Inc., Cary, NC, USA). Data were presented as median (5th percentile, 95th percentile). Kruskal-Wallis test followed by Steel-Dwass analysis or Chi square test was applied when the difference in indices among the 3 groups was evaluated. Comparisons of CRS patients with or without asthma were made using Wilcoxon rank-sum test. For categorical variables, chi-square tests or fisher exact tests were applied. A p value ≤ 0.05 was considered significant when α error was set at 5%.

Spearman rank correlation was used to assess association of sputum periostin with other lower airways variables, and association between upper and lower airways indices. When a ρ value showed < |0.40|, we considered that a correlation between variables might not mean a lot even if a p value was significant.

## Results

### Participant's characteristics

Eighty-four participants (56 CRS patients [20 with and 36 without comorbid asthma] and 28 healthy subjects) were eligible for this study. Characteristics of participants were shown in Table 1. Among patients with CRS, 20 were comorbid with asthma and 19 of which had NPs. Thirty-five patients were sensitized to at least one or more perennial (n = 25) and/or seasonal (n = 29) allergens. Furthermore, 8 patients (5 with and 3 without asthma) previously experienced dyspnoea, cough, and/or chest tightness after taking nonsteroidal anti-inflammatory drugs (NSAIDs) and/or after using toothpaste containing sodium benzoate and paraben. Although we did not perform aspirin challenge test, they were considered as having aspirin-intolerance. Of these 8 patients, 4 (3 with comorbid asthma and 1 without asthma) were sensitized to perennial allergens and 3 with comorbid asthma showed sensitization to seasonal allergens. The proportion of never smokers was significantly higher in healthy subjects than in CRS patients with/without asthma. Meanwhile, other characteristics such as age, sex, and body mass index were similar among CRS patients with/without asthma and healthy subjects. The median duration of sinusitis and asthma was 3 (0.5, 18) years and 5 (0.5, 15) years, respectively. Patients with comorbid asthma showed higher blood eosinophils and total IgE levels than those without asthma, while proportion of atopic predisposition was not affected by comorbid asthma (Table 1). In patients with comorbid asthma, 17 had regularly received inhaled corticosteroids with the mean dose of 605 (295) μg (fluticasone propionate equivalent). According to the GINA 2015 guidelines, 7 patients were classified as step 3, and 10 as step 4. The remaining 3 patients had taken leukotriene receptor antagonists alone and were categorized into step 2. None were taking oral corticosteroids or biologic treatments at enrollment.

**Table 1:** Clinical characteristics of participants.

	All participants (n = 84), except where noted	CRS with asthma (n = 20)	CRS w/o asthma (n = 36)	Healthy subjects (n = 28)	p value
Age, years	60 (30, 76)	60 (41, 70)	61 (34, 77)	59 (27, 74)	0.67
Sex, females, n (%)	35 (42)	5 (25)	14 (39)	16 (57)	0.08^f^
Body Mass Index, kg/m^2^	23.3 (18.5, 28.5)	24.2 (20.0, 27.4)	22.5 (17.8, 31.9)	21.6 (19.1, 26.8)	0.14
Smoking history, never, n (%)	45 (54)	8 (40)	15 (42)	22 (79)	0.005^f^
pack-years	16 (1.5, 61)^a^	15 (0.8, 70)^b^	20 (3., 60)^c^	14 (2.5, 36)^d^	0.88
Aspirin intolerance, n (%)	8 (14)^e^	5 (25)	3 (8)	N/A	0.12^f^
Atopic predisposition, n (%)	35 (63)^e^	14 (70)	21 (58)	N/A	0.56^f^
Blood analyses					
Eosinophils, %^c^	4.5 (0.5, 13.2)	9.0 (3.4, 23.4)	2.8 (0.3, 9.8)	N/A	<0.0001^f^
Serum total IgE, IU/mL^c^	155 (15, 1500)	377 (124, 1052)	69 (12, 1510)	N/A	0.0005^f^
Durations of diseases					
Sinusitis, years	3 (0.5, 18)^e^	2 (0.5, 15)	6.7 (0.7, 22)	N/A	0.31
Asthma, years	–	5 (0.5, 15)	N/A	N/A	–

a n = 39.

b n = 12.

c n = 21.

d n = 5.

e n = 56.

f Values were evaluated by Wilcoxon rank-sum test, chi-square test or Fisher's exact test. The remaining p values were analysed by Kruskal-Wallis test. N/A: not assessed or not applicable. Data were presented as median (5th percentile, 95th percentile) or n (%)

### Comparison of upper airway indices

Table 2, Table 3 show the upper and lower airways characteristics of participants when stratified according to the presence or absence of asthma and NPs, respectively.

**Table 2:** The upper and lower airways characteristics of participants stratified according to the presence or absence of asthma.

	All participants (n = 84, except where noted)	Asthma (n = 20)	W/o asthma (n = 36)	Healthy subjects (n = 28)	p value^a^	p value^b^ A *vs* W/o A	p value^b^ A *vs* H	p value^b^ W/o A *vs* H
**Upper airway indices**								
Open essence scores, points	7 (0, 11)	1 (0, 8)	6 (0, 10)	9 (4, 12)	<0.0001	0.029	<0.0001	0.001
SNOT-22, points	15 (0, 66)	28 (11, 62)	24 (4, 77)	2 (0, 8)	<0.0001	0.99	<0.0001	<0.0001
Lund-Mackay scores, points^c^	12 (4, 21)	15 (10, 23)	10 (4, 20)	N/A	N/A	0.006^e^	N/A	N/A
The presence of nasal polyp, n (%)^c^	38 (50)	19 (95)	19 (53)	N/A	N/A	0.001^e^	N/A	N/A
Eosinophils in nasal polyp, HPF	85 (0, 423)	123 (30, 423)	16 (0, 310)	N/A	N/A	0.003^e^	N/A	N/A
Eosinophils in sinus tissue, HPF^d^	66 (0, 595)	95 (38, 743)	35 (0, 501)	N/A	N/A	0.027^e^	N/A	N/A
**Lower airway indices**								
Sputum periostin, ng/mL^f^	7.1 (0.4, 42.3)	19.4 (4.4, 55.7)	8.6 (0.2, 37.1)	1.6 (0.4, 5.9)	<0.0001	0.019	<0.0001	0.002
eosinophils, %^f^	0 (0, 61)	12 (1, 81)	0 (0, 6)	0 (0, 3)	<0.0001	<0.0001	<0.0001	0.72
neutrophils, %^f^	74 (6, 96)	51 (4, 95)	82 (33, 97)	48 (0, 85)	0.0001	0.021	0.83	0.0001
FeNO, ppb^g^	25.8 (10.9, 80.4)	50.4 (26.7, 91.7)	24.1 (6.5, 79.1)	20.6 (15.3, 34.9)	<0.0001	0.0004	<0.0001	0.36
FEV_1_, % predicted	93.2 (69.8, 119.3)	84.5 (53.1, 102.2)	97.1 (70.5, 121.5)	97.9 (77.7, 119.2)	0.006	0.031	0.005	0.76

A: asthma, w/o A: without asthma, H: healthy subjects, SNOT-22: sino-nasal outcome test-22, HPF: high power field.

a Analyzed by Kruskal-Wallis test.

b Analyzed by Steel-Dwass analysis.

c n = 56.

d Sinus tissue samples were taken from 54 patients.

e Analyzed by Wilcoxon rank-sum test or Fisher's exact test.

f Sputum samples were obtained from 65 participants (31 with CRSwNP, 14 with CRSsNP, and 20 controls).

g FeNO was measured in 83 participants except for one with CRSwNP. Data were presented as median (5th percentile, 95th percentile)

**Table 3:** The upper and lower airways characteristics of participants stratified according to the presence or absence of nasal polyps.

	CRSwNP (n = 38)	CRSsNP (n = 18)	Healthy subjects (n = 28)	p value^a^	p value^b^ wNP vs sNP	p value^b^ wNP *vs* H	p value^b^ sNP *vs* H
**Upper airway indices**							
Open essence scores, points	2 (0, 9)	7 (2, 10)	9 (4, 12)	<0.0001	0.007	<0.0001	0.053
SNOT-22, points	25 (10, 63)	25 (5, 78)	2 (0, 8)	<0.0001	0.99	<0.0001	<0.0001
Lund-Mackay scores, points^c^	15 (5, 23)	7 (4, 13)	N/A	N/A	<0.0001^c^	N/A	N/A
Eosinophils in sinus tissue, HPF^c^	87 (3, 572)	35 (0, 455)	N/A	N/A	0.12^c^	N/A	N/A
**Lower airway indices**							
Sputum periostin, ng/mL^d^	12.0 (0.5, 47.8)	11.2 (1.0, 46.3)	1.6 (0.4, 5.9)	<0.0001	0.91	<0.0001	0.0005
eosinophils, %^d^	2 (0, 74)	0.5 (0, 5)	0 (0, 3)	0.017	0.38	0.020	0.20
neutrophils, %^d^	78 (9, 97)	81 (47, 96)	48 (0, 85)	0.004	0.70	0.0499	0.002
FeNO, ppb^e^	38.1 (14.1, 109.5)	26.8 (6.5, 53.0)	20.6 (15.3, 34.9)	0.001	0.11	0.001	0.31
FEV_1_, % predicted	92.2 (65.0, 114.0)	86.9 (67.9, 121.0)	97.9 (77.7, 119.2)	0.15	0.86	0.19	0.29

wNP: CRSwNP, sNP: CRSsNP, H: Healthy subjects, SNOT-22: sino-nasal outcome test-22, HPF: high power field.

a Analyzed by Kruskal-Wallis test.

b Analyzed by Steel-Dwass analysis.

c Analyzed by Wilcoxon rank-sum test. Data were presented as median (5th percentile, 95th percentile).

d Sputum samples were obtained from 65 participants (31 with CRSwNP, 14 with CRSsNP, and 20 controls).

e FeNO was measured in 83 participants except for one with CRSwNP. Data were presented as median (5th percentile, 95th percentile)

CRS patients showed lower Open Essence scores and higher SNOT-22 scores than healthy subjects, indicating that their olfactory function and QoL were impaired by CRS (Table 2). Particularly, olfactory function was worst in CRS patients with comorbid asthma, followed by those without asthma and healthy subjects in this order (Table 2), while CRS-related QoL was similar between CRS patients with and without asthma (Table 2).

When confined to CRS patients, 38 had NPs. Patients with asthma had significantly higher prevalence of NPs, radiologically more severe CRS, more prominent infiltration of eosinophils in NPs and sinus tissues, as compared with those without asthma (Table 2). These suggest that comorbid asthma would be associated with more severe CRS, as reflected by olfactory dysfunction and upper airway eosinophilia.

When they were stratified according to the presence or absence of NPs (Table 3), patients with CRSwNP showed more significant impairment of olfactory function than those with CRSsNP (Table 3). Additionally, radiological severity was also significantly higher in patients with CRSwNP than in those with CRSsNP (Table 2).

### Comparison of lower airways indices

To evaluate the impact of CRS on the lower airways, inflammatory and functional markers of lower airways were compared among the three groups (Table 2). Sputum periostin levels were highest in CRS patients with comorbid asthma, followed by those without asthma and healthy subjects, in this order. Other Th2-driven biomarkers such as sputum eosinophils and FeNO were significantly higher in CRS patients with comorbid asthma than the other two groups. On the contrary, CRS patients without asthma showed significantly higher sputum neutrophils than those with comorbid asthma and healthy subjects. FEV_1_ values were lower in patients with comorbid asthma than the other groups.

When confined to CRS patients, sputum periostin levels were significantly correlated with sputum eosinophils, and FeNO levels (Table 4), but not sputum neutrophils (Table 4). %FEV_1_ also showed a significant correlation with sputum periostin, though it was weak (Table 4).

**Table 4:** Correlation between sputum periostin and other lower airways indices in CRS patients (n = 45).

	ρ	p value
Sputum eosinophils, %	0.44	0.003
neutrophils, %	−0.08	0.59
FeNO^a^, ppb	0.49	0.001
FEV_1_, % predicted	−0.32	0.0008

Spearman rank correlation test was adopted

a Only one CRS patient with comorbid asthma failed to conduct the NO measurement because of apparatus failure.

When CRS patients were stratified according to the presence or absence of NPs (Table 3), all lower airways indices were similar between these CRS subgroups.

### Association of upper and lower airways indices

Lastly, associations between upper and lower airway indices in CRS patients were investigated. Impaired olfactory function as examined by the Open Essence method showed significant but weak correlation with increased levels of sputum eosinophils (Table 5), while it was unrelated to sputum periostin, or FeNO (Table 5). Meanwhile, the CRS-related QoL examined by SNOT-22 unrelated to any biomarkers of the lower airways (data not shown).

**Table 5:** Correlation between Th2-driven lower airway indices and upper airway indices.

	Sputum periostin, ng/mL^a^		Sputum eosinophils, %^a^		FeNO, ppb^b^	
	ρ	p value	ρ	p value	ρ	p value
Open essence scores, points	−0.08	0.58	−0.35	0.017	−0.22	0.10
Lund-Mackay scores, points	0.18	0.24	0.36	0.014	0.18	0.18
Eosinophils in nasal polyp, HPF	0.45	0.017	0.35	0.056	0.42	0.01
Eosinophils in sinus tissue, HPF^c^	0.31	0.043	0.29	0.062	0.32	0.021

a Sputum samples were obtained from 45 participants (31 with CRSwNP and 14 with CRSsNP).

b FeNO was measured in 55 participants (37 with CRSwNP and 18 with CRSsNP).

c n = 43 for sputum periostin and eosinophils, and n = 53 for FeNO

The infiltration of eosinophils in NPs was significantly correlated with levels of sputum periostin and FeNO, but not eosinophil counts (Table 5). The association between eosinophils in NPs and sputum periostin tended to be significant even when the asthmatics were excluded (n = 14, ρ = 0.43, p = 0.098). All of Th2-driven lower airway biomarkers also showed weak correlations with eosinophils in sinus tissues (Table 5). However, sputum periostin levels and FeNO were unrelated to the radiological severity of CRS, unlike sputum eosinophil counts (Table 5). In addition, sputum neutrophil counts did not correlate with olfactory function, the infiltration of eosinophils in NPs and in sinus tissue or the radiological severity of CRS (ρ = 0.29, p = 0.0504 for olfactory function, n = 31, ρ = −0.09, p = 0.65 for eosinophils in NPs, n = 43, ρ = −0.21, p = 0.17 for eosinophils in sinuses tissue, and n = 45, ρ = 0.04, p = 0.79 for radiological severity of CRS).

Additionally, olfactory function, CRS-related QoL, and eosinophils in NP or sinus tissues were unrelated to FEV_1_ (data not shown).

## Discussion

We have demonstrated, for the first time to the best of our knowledge, that periostin levels are increased in the lower airways, as examined by sputum, of patients with CRS, even when asthma does not coexist. These levels, as well as FeNO levels, were significantly associated with eosinophilia of NPs. Moreover, the impairment of olfactory function was prominent when CRS patients had comorbid asthma. These results strongly indicate the existence of mutual interaction between upper and lower airways, which would affect the pathophysiology of unified airway diseases.

CRS patients showed a significant increase of sputum levels of periostin as compared with healthy controls, but these levels were comparable when the patients were stratified by the presence or absence of NPs. In the gene expression analysis of periostin comparing between NPs and inflamed sinuses of patients with CRSwNP, the expression was five-fold higher in NPs than in inflamed sinuses,^27^ suggesting that NPs are the main source of periostin. On the other hand, eosinophilic NPs were reportedly less prevalent in the Asian than in the Western population; less than 50% of NPs were eosinophilic in Korea.^28^ The same authors also found a significantly higher expression of periostin in eosinophilic than in noneosinophilic NPs.^28^ These results may be consistent with similar sputum levels of periostin between our patients with CRSwNP and those with CRSsNP. We have also demonstrated a significant correlation between eosinophilia of NPs and sputum periostin and FeNO levels, but not sputum eosinophils. This may indicate the association between eosinophilic NPs, which might produce IL-4/IL-13, and the lower airways through naso/sino-bronchial crosstalk. Indeed, the association between sputum periostin levels and the number of eosinophils in NPs tends to be significant when confined to CRS patients without asthma. However, it remains unclear how naso/sino-bronchial crosstalk between upper and lower airways is involved in the upregulation of IL-4/IL-13, if present, in the unified airways.

There is another explanation of increased expression of periostin in lower airways even when asthma does not coexist. In experimental models using rabbits, artificial sinusitis established by a complement fragment could elicit AHR to histamine.^29^ In this report, post-nasal dripping of inflammatory cells and proteins into the lower airway was the most plausible mechanisms for the development of AHR. Indeed, patients with chronic cough and post-nasal drip showed increased levels of sputum neutrophils and TNF-α than normal subjects,^30^ which is line with the present findings of significant increase in sputum neutrophils in CRS patients without asthma (Table 2). However, the link of periostin to neutrophils is less certain than that to eosinophils.^9^ Furthermore, we cannot exclude the possibility that neutrophils may have seemed increased “passively”, and secondary to the lower percentage of eosinophils as compared with CRS patients with asthma.

Anosmia is a major symptom of CRS and it is often refractory to topical corticosteroids.^31^ Eosinophilic inflammation in sinus or NP tissues has been associated with the prevalence and severity of anosmia in CRS patients.^32^^,^^33^ Asthma is also known as one of risk factors of olfactory dysfunction in CRS patients as has been confirmed in this study (Table 2) in addition to aging, the presence of NPs (Table 3), and current smoking.^33^, ^34^, ^35^ Decline in CRS-related QoL is also associated with poor asthma control^36^ and asthma exacerbations that require oral corticosteroids^37^ in CRS patients with comorbid asthma. However, CRS patients with comorbid asthma showed similar preoperative QoL scores assessed by SNOT-20 when compared to those without asthma.^38^ These results are consistent with the present study that olfactory dysfunction was more severe in CRS patients with comorbid asthma than those without asthma, while the impairment of CRS-related QoL was similar between the two groups. Additionally, olfactory dysfunction, but not CRS-related QoL, showed a correlation with sputum eosinophils (Table 5). These suggest that olfactory dysfunction in CRS patients is a potential sign of the existence of eosinophilic inflammation in lower airways and comorbid asthma.

We have firstly demonstrated the relevance of sputum periostin levels in the pathophysiology of unified airway diseases. In the present study, sputum periostin levels were significantly higher in CRS patients with comorbid asthma than those without asthma. This may be the consequence of asthma. Periostin could facilitate not only the recruitment of allergen-induced eosinophils to the lung tissue but also a 5- to 8- fold increase of eosinophil adhesion to fibronectin.^39^ Furthermore, it also activates the migration of IL-5-stimulated eosinophils by binding to CD11b.^40^ These indicate that periostin in the lower airways may be further produced through activated eosinophils under an inflammatory situation like asthma. Some studies have shown the implication of sputum periostin in the pathophysiology of asthma.^12^, ^13^, ^14^^,^^41^ Sputum periostin levels correlated with sputum eosinophilia in patients with severe^12^ and poorly controlled asthma.^14^ Recently, its levels were significantly higher in patients with severe type 2 asthma than in those with mild to moderate asthma.^13^ Furthermore, there were significant correlations of sputum periostin levels with lung function^12^ and sputum specific IgE against *Dermatophagoides pteronyssinus* levels.^41^ Hence, sputum periostin might be a potential biomarker of patients with severe type 2 asthma who are expected to show good response to biologics.^42^ Sputum periostin levels of our subjects (0.1–70.7 ng/mL) showed similar range to previous studies (0.1–152 ng/mL),^12^, ^13^, ^14^^,^^41^ indicating the validity of our measurement.

We would like to point out some limitations to our study. First, 33 of 56 CRS patients had past smoking history. Although smoking is one of the most important factors of the development of CRS, the development of the lower airway inflammation may be affected by past smoking rather than CRS-related factors such as naso/sino-bronchial crosstalk and post-nasal drips. However, the influence of smoking on sputum periostin levels has not been confirmed yet; one study of poorly-controlled asthma showed similar periostin levels between never smokers and past smokers.^14^ Second, not all of our asthmatic patients had very severe disease that may require biologics. On the contrary, CRS of all patients were intractable to pharmacological treatments and had an indication of ESS. Therefore, our findings may not be applicable for milder CRS patients and CRS patients with comorbid more severe asthma. Third, we did not measure total protein in sputum because of the lack of samples. In the study presented here, sputum periostin levels were not corrected by total protein content in the previous studies. However, we cannot preclude the possibility that the content of total protein in collected sputum samples may influence the measurement of periostin levels. To address these problems, studies of larger cohorts, including both severe asthma and non-severe CRS, are needed to further determine the pathophysiological link between upper and lower airways, particularly the association of olfactory function with sputum periostin levels, eosinophilia and FeNO levels.

In conclusion, we have demonstrated that periostin levels and inflammatory cells such as eosinophils and neutrophils in the lower airways are increased in patients with CRS, suggesting the presence of mutual interactions between upper and lower airways even if asthma does not coexist. Eosinophilic NPs may be associated with Th2-driven inflammation of the lower airways through naso/sino-bronchial crosstalk and post nasal drips, indicating that eosinophilic NPs may be related to the pathophysiology of unified airway diseases. Moreover, olfactory dysfunction could be a potential indicator of eosinophilic inflammation in lower airways and asthma. Further longitudinal studies are necessary to clarify how mutual interaction between upper and lower airways affects the pathophysiology of unified airway diseases.

## A declaration of all sources of funding for the research

This study was supported in part by research grants from Novartis Pharma, Japan; Tanabe Mitsubishi pharma, Japan; and KOBAYASHI FOUNDATION, Japan.

## Consent for publication and funding

Y.K. reports research grants from Novartis Pharma, and Tanabe Mitsubishi pharma for the submitted work, and grants from MSD and Kyowa-Kirin corporations outside the submitted work. M.S. reports research grants from Kobayashi Foundation for the submitted work. S. F. reports personal fees from AstraZeneca, personal fees from Eli Lilly Japan outside the submitted work. H.O reports research grant from Boehringer Ingelheim outside the submitted work. K.M. reports personal fees from Pfizer and Chugai Pharmaceutical outside the submitted work. T.O reports personal fees from AstraZeneca, Eli Lilly Japan, Taiho Pharmaceutical, Pfizer, Chugai Pharmaceutical, MSD, Daiichi Sankyo, and Asahi Kasei Pharma, and research grants and personal fees from Kyowa Hakko Kirin, Boehringer Ingelheim, Ono Pharmaceutical, and Novaltis outside the submitted work. K.I. reports research grants from Shino-Test Corporation for the submitted work. M.T reports research grant from Pfizer outside the submitted work. A.N. reports personal fees from Astellas, AstraZeneca, Kyorin, GSK, and Boehringer Ingelheim, and research grants from Astellas, Kyorin, Boehringer Ingelheim, Novartis, MSD, Daiichi Sankyo, Taiho, Teijin, Ono, Takeda, Maruho Pharmaceutical outside the submitted work.

## Availability of data and materials

Not applicable.

## Conflict of interest

None.

## Ethics approval and consent to participate

This study was approved by the Ethics Committee of Nagoya City University (Number 1165) and was registered on the UMIN Clinical Trials Registry (Registry ID UMIN000018672). Written informed consent was obtained from all participants.

## Author's contribution

YK: established the conception of the whole study, and contributed to the performance of diagnostic tests, the collection of data, the recruitment of patients, disease diagnosis and management, the acquisition and interpretation of data, and drafting the manuscript. KF, NT, RK, HN and JY contributed to the performance of diagnostic tests, the collection of data, and the acquisition and interpretation of data. YN, TA, and MT contributed to the recruitment of patients, disease diagnosis and management, and revision of the manuscript. YO contributed to assess the radiological severity of CRS. AM made specimens and assessed the infiltration of eosinophils in upper airway tissues. SF, HO, KM, YI, and TO contributed to the diagnostic tests, the collection of data, and management of patients. KI, SO, and JO carried out the measurement of periostin. MS and AN contributed to the recruitment of patients, disease diagnosis and management, interpretation of data, and revision of the manuscript.
